# Prognostic importance of harvested lymph node number, metastatic lymph node number, and lymph node ratio in surgically managed laryngeal squamous cell carcinoma

**DOI:** 10.1016/j.bjorl.2020.07.012

**Published:** 2020-09-10

**Authors:** Mehmet Akif Abakay, Selçuk Güneş, Filiz Gülüstan

**Affiliations:** a Health Science University Bakırköy Dr Sadi Konuk Research and Training Hospital, Istanbul, Turkey; b Memorial Hizmet Hospital, Istanbul, Turkey

**Keywords:** Prognosis, Survival, Neck dissection, Metastase, Lymph node

## Abstract

**Introduction:**

Lymph node metastasis is a well-known prognostic factor for laryngeal carcinoma. However, current nodal staging systems provide limited information regarding prognosis. Additional parameters should be considered to improve prognostic capacity.

**Objectives:**

To assess the prognostic values of metastatic lymph node number, ipsilateral/contralateral harvested lymph nodes, and lymph node ratio in patients undergoing surgical treatment of laryngeal squamous cell carcinoma.

**Methods:**

Seventy-four patients diagnosed with laryngeal squamous cell carcinoma primarily managed surgically were included in this study. The patients’ pathological and survival data were obtained from their medical records. The effects of harvested lymph nodes and lymph node ratio on disease-free survival, disease-specific survival, and overall survival were analyzed.

**Results:**

Ipsilateral, contralateral, and bilateral evaluations of harvested lymph nodes showed no significant associations with prognosis. Lymph node ratio was significantly associated with overall survival when evaluated bilaterally. Metastatic lymph node number showed more suitable stratification than TNM classification.

**Conclusions:**

Metastatic lymph node number and bilateral lymph node ratio parameters should be taken into consideration to improve the prognostic capacity of TNM.

## Introduction

Laryngeal Carcinoma (LC) is one of the most common head and neck cancers; it can be managed with surgery and/or radio/chemoradiotherapy. Patients are classified according to the American Joint Committee on Cancer (AJCC) system, and treatment is mainly planned according to the primary Tumor (T), lymph Node (N), metastasis, and distant Metastasis (M) status. Improvements in treatment modalities are necessary to reduce the rates of mortality and morbidity.

Lymph node metastasis is a well-known prognostic factor for laryngeal carcinoma.^1^ The TNM system N status provides only limited information regarding prognosis. Therefore, to improve its prognostic ability, new parameter investigations have been underway for some time. A recently investigated parameter comprises Harvested Lymph Nodes (HLN). Although conflicting results have been reported, Divi et al.^2^ reported that resection of < 18 HLNs was associated with poor prognosis in patients with Node-positive (N+) head and neck cancer. In addition, an N classification based on the number of Metastatic Lymph Nodes (MLN) examined has been proposed instead of the AJCC N system for patients with head and neck cancer.^3^, ^4^ The Lymph Node Ratio (LNR), which is defined as MLN/HLN, is another newly investigated parameter that exhibits prognostic potential in patients with laryngeal cancer.^3^, ^4^, ^5^, ^6^, ^7^, ^8^

However, to the best of our knowledge, there have been no studies comparing the ipsilateral and contralateral neck in terms of HLN and LNR. Therefore, the present study investigated whether evaluation of HLNs and LNR on the ipsilateral/contralateral or bilateral sides yielded useful information regarding prognosis. In addition, this study evaluated MLNs and compared them with the LNR and HLNs.

## Materials and methods

Ethical approval for this study was obtained from the local ethics committee (approval nº 2019/460). Patients diagnosed with laryngeal squamous cell carcinoma who underwent surgical treatment between January 2010 and October 2018 at Bakırköy Dr. Sadi Konuk Teaching and Research Hospital were included in this study. Patients who underwent laryngectomy with neck dissection were reviewed retrospectively. Patients who underwent previous neck dissection or previous radiation therapy, who had multiple primary lesions, stage 4c, positive surgical margin, and who were followed up for < 1 year were excluded from the study. In total, 74 patients were eligible for inclusion in the study. The patients’ pathological parameters (e.g., surgical margins, tumor localization, grade, differentiation, perineural invasion, vascular invasion, ipsilateral and contralateral HLN, and MLN) and follow-up information (e.g., visit times, recurrence, or death) were recorded.

Selective neck dissection was performed unilaterally or bilaterally according to tumor location and preoperative evaluation. Level II–IV (Level V dissection was also performed in patients with subglottic extension >1 cm and direct invasion) lymph nodes were resected in an en bloc manner, as recommended by the American Head and Neck Society. Adjuvant treatment was administered in accordance with the postoperative pathology results. Patients were classified in accordance with the 2018 AJCC TNM classification. Adjuvant radiotherapy was evaluated in terms of adverse risk factors, such as perineural invasion, lymphovascular invasion, positive metastatic lymph nodes, and T3–T4 tumors. Recurrences were detected either pathologically or radiologically.

### Survival parameters

Disease-Free Survival (DFS: time between the date of curative surgery to the first recurrence date or last follow-up, Disease-Specific Survival (DSS: time between the date of curative surgery to the date of the last follow-up or death due to disease), and Overall Survival (OS: time between the date of curative surgery to the date of the last follow-up or death of any cause) were calculated using the Kaplan–Meier method.

### Statistical analysis

Receiver Operating Characteristic (ROC) analysis was performed for HLN, MLN, and LNR for the ipsilateral neck, contralateral neck, and bilateral sides. In addition, a cutoff value of 18 for HLN was set to allow comparison with the literature.^2^ MLNs were classified as Group 1, 0 MLNs; Group 2, 1 MLN; Group 3, 2–4 MLNs; Group 4 ≥ 5 MLNs.^9^ Pearson’s correlation coefficient was used to detect associations between lymph node parameters and other pathological parameters. Logistic regression analysis was performed for univariate analysis. Multivariate analysis was performed using the Enter and Forward methods for factors with *p* < 0.1. In all analyses, *p* < 0.05 was considered to indicate statistical significance.

## Results

The study population consisted of 70 (95%) men and 4 (5%) women with an average age of 59.63 ± 8.52 years. The mean follow-up period, recurrence time, and OS were 37.08 ± 21.82 months, 35 ± 21 months, and 37.86 ± 21.73 months, respectively. During the follow-up period, 12 (16%) recurrences and 16 (21%) deaths (7 [9%] of which were due to recurrence) occurred. The pathological data of the patients are presented in Table 1. The numbers of ipsilateral and contralateral HLNs were 21 ± 9 (range, 4–43) and 20 ± 8 (range, 5–44), respectively; the numbers of ipsilateral and contralateral MLNs were 1 ± 3 (range, 0–24) and 1 ± 2 (range, 0–12), respectively. The total number of HLNs was 39 ± 16 (range 9–87); the ipsilateral, contralateral, and bilateral LNR were 0.05 ± 0.11 (range, 0–0.77), 0.02 ± 0.06 (range, 0–0.43), and 0.03 ± 0.07 (range, 0–0.34), respectively.

**Table 1:** Pathological features.

Parameter		n	%
T	1	14	18.9
	2	22	29.7
	3	28	37.8
	4	10	13.5
N	0	48	64.9
	1	6	8.1
	2	19	25.7
	3	1	1.4
Stage	1	13	17.6
	2	16	21.6
	3	18	24.3
	4	27	36.5
Tumor localization	Supraglottic	24	32.4
	Glottic	27	36.5
	Subglottic	2	2.7
	Transglottic	21	28.4
Differentiation	Well	11	14.9
	Moderate	45	60.8
	Poor	18	24.3
Perineural invasion	Absent	58	78.4
	Present	16	21.6
Lymphovascular invasion	Absent	55	74.3
	Present	19	25.7
MLN	0	48	64.9
	1	14	18.9
	2–4	5	6.8
	≥ 5	7	9.5
Ipsilateral LNR	≤ 0	51	68.9
	> 0	23	31.1
Bilateral LNR	< 0.0286	52	70.3
	≥ 0.0286	22	29.7

**Figure 1:**
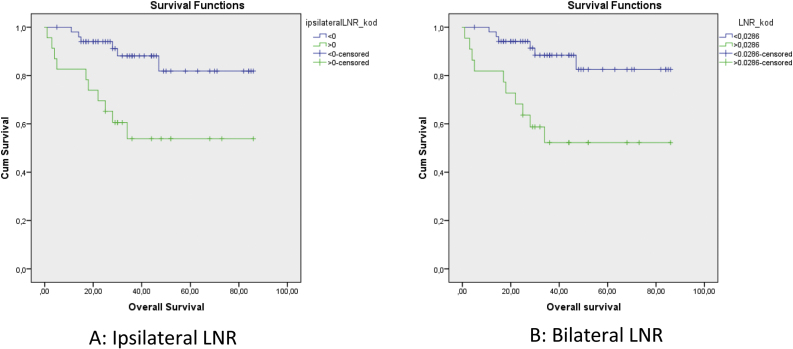
Kaplan–Meier curves for ipsilateral LNR (A), and bilateral LNR (B).

**Figure 2:**
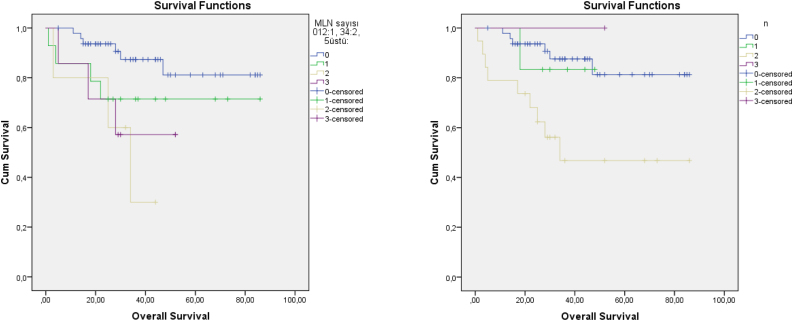
Kaplan–Meier curves for MLN grading system (left) and AJCC N classification (right).

The cut-off values calculated in ROC analysis are shown in Table 2. ROC analysis of the associations of ipsilateral HLNs and LNR, contralateral HLNs and LNR, and bilateral HLNs and LNR with DFS, DSS, and OS showed that ipsilateral and bilateral LNRs were significantly associated with OS. In addition, we classified N status as N− and N+ because the ipsilateral LNR cut-off value was calculated as 0. The log-rank test indicated that OS was significantly associated with N status (*p* = 0.011), N−/N+ (*p* = 0.01), lymphovascular invasion (*p* = 0.02), MLN (*p* = 0.036), ipsilateral LNR (*p* = 0.003), and bilateral LNR (*p* =  0.002). Kaplan–Meier analysis results for OS are shown in Figure 1, Figure 2. In addition, perineural invasion showed significant associations with OS, DSS, and DFS (*p* < 0.05).

**Table 2:** Cut-off values obtained by ROC analysis (with p-values for LNR).

	DFS	DSS	OS
Ipsilateral HLN	29	31	16
Contralateral HLN	23	16	27
Bilateral HLN	52	40	22
Ipsilateral LNR	0.0345 (*p* = 0.078)	0.0588 (*p* = 0.183)	0 (*p* = 0.008**)**
Contralateral LNR	0.0294 (*p* = 0.147)	0.0526 (*p* = 0.643)	0.0294 (*p* = 0.182)
Bilateral LNR	0.0357 (*p* = 0.079)	0.0377 (*p* = 0.204)	0.0286 (*p* = 0.012)

Multivariate analyses for OS were performed using N− vs. N+ classification, N status, lymphovascular invasion, ipsilateral LNR, and bilateral LNR and MLN parameters. Total LNR exhibited a significant association with OS (hazard ratio [HR]: 4.381, 95% Confidence Interval [95% CI]: 1.590–12.068, *p* = 0.004). Analyses for DFS were performed using ipsilateral LNR, bilateral LNR, perineural invasion, and N−/N+ parameters. Perineural invasion showed a significant association with DFS (HR = 3.838, 95% CI: 1.236–11.916, *p* = 0.020). Regression analysis for DSS was performed using perineural invasion as the sole parameter (HR = 9.227, 95% CI: 1.799–47.842, *p* =  0.008). The associations of the patients’ pathological data with ipsilateral and bilateral LNRs are shown in Table 3.

**Table 3:** Distribution of pathological features according to LNR.

	Ipsilateral LNR			Bilateral LNR		
	≤0	>0	*p*	<0.0286	≥0.0286	*p*
T1	13	1	0.032	13	1	0.020
T2	17	5		18	4	
T3	16	12		15	13	
T4	5	5		6	4	
N0	48	0	< 0.001	47	1	< 0.001
N1	2	4		4	2	
N2	1	18		1	18	
N3	0	1		0	1	
Stage 1	13	0	< 0.001	13	0	< 0.001
Stage 2	16	0		16	0	
Stage 3	16	2		16	2	
Stage 4	6	21		7	20	
Supraglottic	16	8	0.250	17	7	0.104
Glottic	22	5		22	5	
Subglottic	2	0		2	0	
Transglottic	11	10		11	10	
Well-differentiated	8	3	0.040	8	3	0.025
Moderately differentiated	35	10		36	9	
Poorly differentiated	8	10		8	10	
Perineural invasion absent	42	16	0.236	42	16	0.539
Perineural invasion present	9	7		10	6	
Lymphovascular invasion absent	48	7	< 0.001	48	7	< 0.001
Lymphovascular invasion present	3	16		4	15	

## Discussion

Univariate analysis indicated that ipsilateral LNR, bilateral LNR, N status, N−/N+, MLN, and lymphovascular invasion showed significant associations with OS. Multivariate analysis showed that bilateral LNR was the only factor that affected OS. Perineural invasion showed significant associations with DFS and DSS in both univariate and multivariate analysis. However, for HLN, neither ROC cutoffs nor the previously reported value of 18 showed associations with DFS, DSS, or OS. T, N, stage, differentiation, and lymphovascular invasion were associated with both ipsilateral LNR and bilateral LNR. Contralateral LNR and contralateral HLN showed no significant associations with survival.

Harvesting adequate numbers of lymph nodes during neck dissection is of crucial importance for both N0 and N + laryngeal carcinomas. During neck dissection, well-described levels should be cleared with all lymphatic pathways in laryngeal carcinoma.^10^ However, there are conflicting results in the literature regarding the number of lymph nodes for adequate resection. Divi et al.^2^ reported that resection of ≥ 18 LNs was associated with improved OS and locoregional control in patients with surgically managed N + head and neck cancers. Some studies of patients who were both N − and N + investigated laryngeal cancer and other head and neck sites; those studies found that HLN numbers were associated with OS.^3^, ^11^, ^12^ Roberts et al.^9^ investigated HLNs in patients with N0 laryngeal carcinoma and found no association with survival. In addition, Bötcher et al.^13^ investigated the association between HLNs and OS in patients with N0/N + laryngeal carcinoma and found no significant association. Our study included patients with both N0 and N + laryngeal carcinomas. We used two different cutoff values (ROC 16, 27, and 22 for ipsilateral, contralateral, and bilateral carcinomas, respectively; 18 for all carcinomas); none showed a significant association with OS. Our results were compatible with previous findings because our study population included patients with both N− and N+ carcinomas; moreover, there was no apparent association between HLN number and survival in patients with surgically managed laryngeal carcinoma. Another important factor is that lymph node number can be affected by pathological processing, as well as pathologist and surgeon experience. These aspects should be considered when evaluating the effects of HLNs on prognosis.^2^

Recent studies showed that MLN number had greater prognostic value than the current nodal staging systems. Ho et al.^3^ recommended a new N staging system that classified patients according to the number of MLNs and extranodal extension; an evaluation of 8351 patients revealed that their staging system was more informative than the current 8th AJCC N staging system. Choi et al.^4^ also reported that the MLN and extranodal extension-based staging system had better survival predictive value when compared to the AJCC system. Roberts et al. compared three nodal classification systems in 12,417 patients with head and neck cancer — (1) 8th AJCC N staging, (2) Staging according to LNR, and (3) Staging according to MLNs; notably, staging according to MLNs (Group 1, 0; Group 2, 1; Group 3, 2–5; and Group 4, ≥5) had prognostic value superior to that of the other systems.^9^ We used the same MLN staging numbers used by Roberts et al.,^9^ but were unable to evaluate extranodal extension. Our Kaplan–Meier curve showed similar results, especially after 2 years. MLNs showed better prognostic stratification than the current N staging system. In addition, as shown in Fig. 2, the level 3N status of the TNM and MLN system did not show the expected linearity. Node status for N3 is described as ≥6 cm in the 7th edition of the AJCC, which caused difficulty in terms of deciding between multiple lymph node metastases or single lymph nodes >6 cm. Therefore, some studies did not evaluate N3 status.^14^ We had only one patient at N3. Five patients were grade 5 in terms of MLN 3 grade, which produced more predictable curves.

Sano et al.^1^ investigated the prognostic importance of LNRs in 67 patients with head and neck squamous cell carcinoma for both OS and locoregional recurrence-free survival. Imre et al.^15^ investigated the prognostic importance of LNR in 101 patients with pN + laryngeal squamous cell carcinoma. They reported that LNR ≥ 0.09 and ≥ 4 MLNs were associated with OS and DFS. Süslü et al.^6^ reported that LNR > 0.04 was associated with poor prognosis; thus, it could be considered an indication for postoperative radiotherapy treatment in patients with N + head and neck cancer. Choi et al.^5^ investigated 156 patients with N + laryngeal squamous cell carcinoma and found that lymph node density was more closely associated with cancer-specific mortality, compared to pT, pN, extralaryngeal spread, and thyroid cartilage invasion. We investigated ipsilateral LNR, contralateral LNR, and bilateral LNR as unique parameters; we found no associations between contralateral LNR and OS, DFS, and DSS. Both ipsilateral LNR and bilateral LNR showed a significant association with OS in univariate analysis. However, multivariate analyses showed that bilateral LNR was the only parameter significantly associated with OS. Our results indicate that evaluation of LNRs in the bilateral neck may be more valuable than ipsilateral or contralateral evaluations alone.

The indications for bilateral neck dissections are controversial. Therapeutic bilateral neck dissection is recommended in patients with a contralateral clinically metastatic lymph node. Elective contralateral neck dissection is not recommended for glottic tumors, regardless of ipsilateral positive nodes.^16^ Elective contralateral neck dissection is recommended for supraglottic tumors if the tumor crosses the midline, T3 and T4, in combination with suspicion of extracapsular invasion.^16^ Moreover, the presence of epiglottic invasion and clinically positive lymph nodes have been reported to increase the likelihood of bilateral lymph node metastasis.^17^ The significant association between bilateral LNR and OS, which is the most important result of this study, suggest a need to re-evaluate the indications for bilateral neck dissection in patients undergoing laryngeal cancer resection. Further studies in larger populations are required to confirm our conclusions.

The difference between evaluation of MLNs and LNR is important because sufficient lymph node resection is also important for locoregional control and reflects the quality of surgery; evaluation of resection by means of LNRs increases the prognostic value of surgery.^1^ This presumably explains why bilateral LNR was a significant factor, rather than MLN, in multivariate analyses in the present study. In addition, investigations of known predictors of adverse tumor pathological features (e.g., T, N, stage, differentiation, lymphovascular status, and LNR) showed significant associations with survival.

This study had several limitations. We could not evaluate associations with extranodal extension or smoking and alcohol status because of the retrospective study design. In addition, this study only reflected survival of patients with surgically managed laryngeal carcinoma because clinical N status and pathological N status may differ. Nevertheless, our findings are valuable and are the first to show that bilateral LNR status has better prognostic predictive value than either ipsilateral or contralateral LNR status alone.

## Conclusion

Prognostic markers are important in treatment planning and follow-up of patients with laryngeal carcinoma. There have been major changes in otorhinolaryngology with the most recent changes to the TNM system. It may be necessary to add bilateral LNR status to increase the prognostic predictive capability of the N system in laryngeal cancer.

## Funding

This research did not receive any specific grants from funding agencies in the public, commercial, or not-for-profit sectors.

## Conflicts of interest

The authors declare no conflicts of interest.
